# Effectuating worthy medical training without neglecting health services in Mexico

**DOI:** 10.1016/j.lana.2024.100739

**Published:** 2024-04-09

**Authors:** Diego Ramonfaur, Mauricio Torres-Martínez

**Affiliations:** a Cleveland Clinic, Department of Internal Medicine, Cleveland, OH, USA; b Tecnológico de Monterrey, Escuela de Medicina, Monterrey, Mexico

Hundreds of rural clinics run by medical students in Mexico are the best access to healthcare for millions of people.^1^^,^^2^ Here, students are often restricted to working for 1 year as underpaid government health employees as a requirement to become licensed physicians; an easy way to palliate the severe healthcare budget constraint.^3^ This framework lowers costs of care by millions of dollars annually, since medical students who run these centres are paid less than minimum wage,^4^ and allows access to healthcare at a very low cost. However, this may in turn backfire and increase costs of care,^5^ as the quality of these services is suboptimal at best given students deliver poorly supervised medical care despite lack of experience.^2^ Employing students as quasi-professionals raises significant quality-of-care questions. The under-supervision of these student-run clinics compromises the quality of healthcare delivery and places students at risk of malpractice, which ultimately affects patients. Every year, students report threats, sexual and psychological abuse. Some have been murdered while on service, particularly at rural clinics.^6^, ^7^, ^8^ Women may be disproportionately affected.^9^ In turn, universities appear to take on a passive role to address this despite the risks. There is an urgent need to rethink this dimension of healthcare in Mexico with a social justice perspective: How to improve social service conditions while maintaining access to healthcare at reasonable costs?

The Mexican Institute of Social Security and Welfare (IMSS-Bienestar) is the government agency that provides healthcare coverage to those without Social Security benefits. States politically unaffiliated to the federal government party opted to self-administer services to underserved populations, magnifying disparities in access by state. This initiative has devolved to create more unsatisfactory student-run clinics to cover rural populations that would otherwise have no access to care.

There are three central problems to this status quo:

(1) Poor funding results in understaffing, limited access to basic resources, and no continuing education for students.
(2) The operational management of these clinics is increasingly challenging as more clinics are built and more people seek care.
(3) Medical students operating these centres are exposed to unacceptable danger while on service.

Service provided by students at these clinics must improve, although there is no easy solution. Centralizing healthcare is an attractive alternative, aiming to remove most rural clinics and enhance suburban centres to serve these populations with the intention of strengthening primary health care. This approach provides four advantages:

(1) Enhanced security, access, and resources for medical students, staff, and providers.
  a. Students may have better security by working in larger centres with other providers and being able to live at the centre with other staff or commute daily from urban areas instead of living unaccompanied in rural communities.
(2) Logistics and governance advantage.
  a. Administrators may focus on a single centre rather overseeing several centres, improving quality control.
(3) Economic leverage.
  a. Funding one large centre may be more feasible than funding many smaller centres.
(4) Wider scope of coverage.
  a. More convenient access to healthcare for villages that previously lacked any form of health-related services.

This proposition comes with challenges. Transportation to and from rural villages to the assigned health centre must be guaranteed for timely medical attention. This will require infrastructure, acquisition of means of transportation, and trained personnel to operate the system. Effective mechanisms of communication and community outreach may mitigate this limitation and should be considered in the form of telehealth, which may reduce the number of visits needed. The centralization of health is a promising measure,^10^ taking into account student safety, wellbeing, and continuing education. Another consideration is the likely reluctance of the general population against this initiative, for which appropriate stakeholder and community engagement is imperative. Collaboration with leaders of these villages to understand beneficiary perspectives and concerns is needed to implement a model that benefits both sides. The threshold to consider a suburban centre is not defined. Geographic conditions that would favour this model encompass an accessible area accessibly via highways from urbanized cities, while being within reasonable driving distance from the villages it aims to serve. To further characterize the healthcare service access in the current era, quantitative and qualitative studies are needed to appropriately design interventions.

Mexico faces severe healthcare constraints. The social service is an outdated policy that has a complex interplay with rural health in Mexico. Rethinking medical training during the social service is imperative to the wellbeing of patients and providers. This proposal suggests centralizing rural clinics into suburban centres to ease administrative challenges and enhance safety and education of healthcare personnel, considering the needs and rights of the population served. We acknowledge the need of a pilot project to test feasibility of the model and provide pragmatic evidence for large-scale implementation.

## Contributors

DR: Idea conceptualization and design of the manuscript, realized extensive literature review, and co-wrote and edited the manuscript.

MT: Provided first-hand insight into the Mexican public healthcare system, realized extensive literature review, and drafted the manuscript.

## Declaration of interests

The authors declare no competing interests.
